# Molecular characterization of hepatitis c virus in multi-transfused Colombian patients

**DOI:** 10.1186/1743-422X-9-242

**Published:** 2012-10-23

**Authors:** Diana di Filippo, Fabian Cortes-Mancera, Mauricio Beltran, Maria Patricia Arbelaez, Sergio Jaramillo, Juan Carlos Restrepo, Gonzalo Correa, Maria-Cristina Navas

**Affiliations:** 1 Grupo de Gastrohepatologia, Sede de Investigacion Universitaria (SIU), Universidad de Antioquia, Carrera 53 # 61-30, Laboratorio 434, Torre 2, Medellin, Colombia; 2 Facultad de Ciencias Exactas y Aplicadas, Instituto Tecnologico Metropolitano (ITM), Institucion Universitaria adscrita a la alcaldia de Medellín, Medellin, CO-549 59, Colombia; 3 Coordinacion Red Nacional de Bancos de Sangre, Instituto Nacional de Salud, Bogota, Colombia; 4 Grupo de Epidemiologia. Facultad Nacional de Salud Publica, Universidad de Antioquia, Medellin, Colombia; 5 Hospital Pablo Tobon Uribe, Calle 78B # 69-240, Medellin, Colombia

## Abstract

**Background:**

Hepatitis C virus (HCV) infects 170 million persons worldwide and is a public health problem. Considering that HCV is principally transmitted by exposure to infected blood, multi-transfused patients constitute one of the most important risk groups in developing countries. To explore the dynamics of this infection in Colombia, we performed a study to determine the genotypes of HCV in a cohort of multi-transfused patients.

**Results:**

The serum samples from patients positive for anti-HCV were evaluated for HCV RNA by nested-PCR of the 5’untranslated region (5’UTR). Viral genotype was determined by RFLP and/or automated sequencing. HCV subtype 1b was found in eight cases (66.7%) and subtype 1a in two cases (16.7%); seven isolates of subtype 1b were obtained from patients who had received the first transfusion before 1986. Either genotypes 2b (8.3%) or 3a (8.3%) were found in the remaining positive specimens.

**Conclusions:**

This is the first HCV genotyping study developed in multi-transfused patients in Colombia where HCV subtype 1b was the most prevalent. The mutation G235A in the 5’UTR of three isolates generated an additional restriction site and an RFLP pattern different from those previously described for genotype 1.

## Background

HCV infection is a global public health problem worldwide. The virus persists in 60 to 85% of infected persons and can lead to end-stage liver diseases and death. Indeed HCV is a principal cause of cirrhosis and liver cancer and a major indication for liver transplantation [1,2].

HCV is a single-stranded RNA virus classified into the family *Flaviviridae,* genus Hepacivirus [3]. Six major genetic groups and numerous subtypes have been described with different geographical distributions, transmission routes and response to antiviral treatments [2,4-7]. Subtypes 1a, 1b, 2b and 3a are the most prevalent [8,9]; subtype 1b is predominantly identified among individuals with history of blood transfusions ; subtypes 1a and 3a are becoming more prevalent in former intravenous drug users (IDU) [10-13].

In developed countries, voluntary blood donation, blood donor education, history-based donor selection and universal blood donor laboratory screening have resulted in improved blood safety and reduced residual risk for transfusion-transmissible infections (TTI), including HCV [14,15]. However, TTI are still a public health problem in developing countries [16].

A cross-sectional study carried out in Colombia showed that the overall prevalence of HCV infection among 500 multi-transfused Colombian patients was 9%; the main risk factors were being a patient with hemophilia, receiving transfusions before 1995 and having received ≥ 48 units of blood components [17].

To better understand the epidemiology of HCV in Colombia, we characterized the HCV genotypes and subtypes in this cohort of multi-transfused patients.

## Results and discussion

The study population included 45 patients with HCV antibodies from the cohort of the cross-sectional study of multi-transfused patients (33 males, 12 females, median age 50 years, median age of first transfusion 14 years). This study was carried out in four hospitals in the two largest cities in Colombia, Bogota and Medellin.

The HCV genome was detected by 5’UTR nested RT-PCR in 12 (26.6%) of the 45 samples obtained from anti-HCV positive multi-transfused patients. The yield rate could be due to viral clearance, low viral load and/or the long period of sample storage. A similar percentage, 28.8%, of HCV genome detection in anti-HCV positive samples was reported in other study [18]; moreover the probability of unsuccessfully PCR amplification is associated with viral loads <100 copies/ml [8].

Ten of the 12 patients (83.3%) received the first transfusion before 1993, the date when testing for HCV in blood transfusions become mandatory in Colombia [17]. Clearly, the risk of HCV infection diminished dramatically in the cohort after 1995. Additionally, all the patients with hemophilia had received cryoprecipitate and 3 of them had also received whole blood (Table 1); indeed the transfusion of whole blood was identified as a risk factor in the multivariate analysis of the cohort [17].

**Table 1:** HCV genotype distribution in Colombian multi-transfused patients

**Code of samples**	**Genotype**	**Number of transfused units/Blood components**	**Year of the first transfusion**	**Diagnostic category**
HCV COL_208	1a	9114*	1950	Hemophilia
HCV COL_188	1b	80*	1960	Hemophilia
HCV COL_178	1b	42*	1962	Hemophilia
HCV COL_173	1b	1992	1972	Hemophilia
HCV COL_XX6	1b	2710	1974	Hemophilia
HCV COL_172	1b	1344	1975	Hemophilia
HCV COL_175	1a	1704	1980	Hemophilia
HCV COL_478	1b	11	1951	Acute bleeding
HCV COL_224	1b	20	1986	Hemodyalisis
HCV COL_278	3a	10	2002	Hemodyalisis
HCV COL_120	2b	10	1998	Oncologic disease
HCV COL_XX1	1b	12	2002	Oncologic disease

*Including whole blood.

These results are consistent with the risk of receiving an HCV positive blood component in Colombia estimated by 1994 (25.4 / 10.000 donations) and the HCV screening coverage in blood donors reported in the same period (67%). However, the screening coverage in 1997 was 100% and consequently this risk decreased to 0 / 10.000 donations that year. This data demonstrates the value of direct and indirect strategies to improve transfusion policy [16,19]

**Figure 1:**
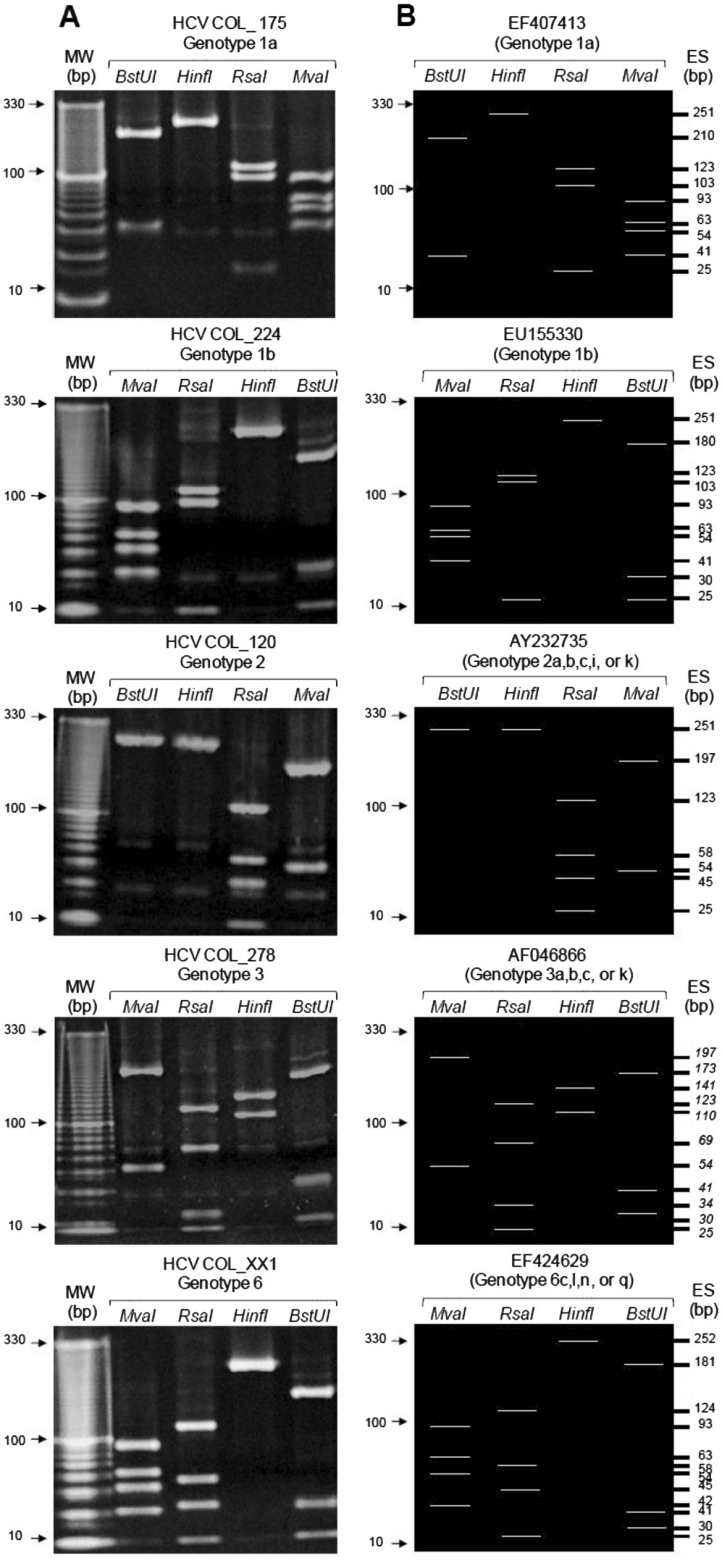
**Identification of patterns obtained by restriction endonuclease digestion of HCV 5′UTR sequences. WM:** Molecular Weight marker 10 bp ladder. **A**. Restriction patterns of samples generated by RFLP. **B**. *In silico* restriction patterns of Genbank sequences generated by Bioedit software; Genbank accession numbers (EF407413, EU155330, AY232735, AF046866, EF424629). **ES**: Expected size of digested fragments.

To analyze the distribution of the different HCV genotypes in this population, we first performed an analysis by RFLP. A comparison among RFLP restriction patterns obtained and those generated by bioinformatics with GenBank sequences allowed the identification of five isolates as subtype 1b, two as subtype 1a, one as genotype 2 and one as genotype 3. In some samples, the viral subtype could not be established by this method due to the similarity between patterns. Unexpectedly, three isolates (HCVCOL_172, 478, XX1) showed a restriction pattern similar to that of genotype 6 (Figure 1).

To verify the results obtained by RFLP, the PCR products of eight isolates (HCVCOL_175, 224, 120, 278, XX6, 172, 478 and XX1) were directly sequenced using primers 940 and 211. Analysis of these sequences by BLAST made it possible to confirm the subtypes 1a and 1b, and to determine the subtypes 2b and 3a. Moreover, the three isolates with the unexpected RFLP pattern (HCVCOL_172, 478 and XX1) were identified as genotype 1 with a similarity of 98%.

**Figure 2:**
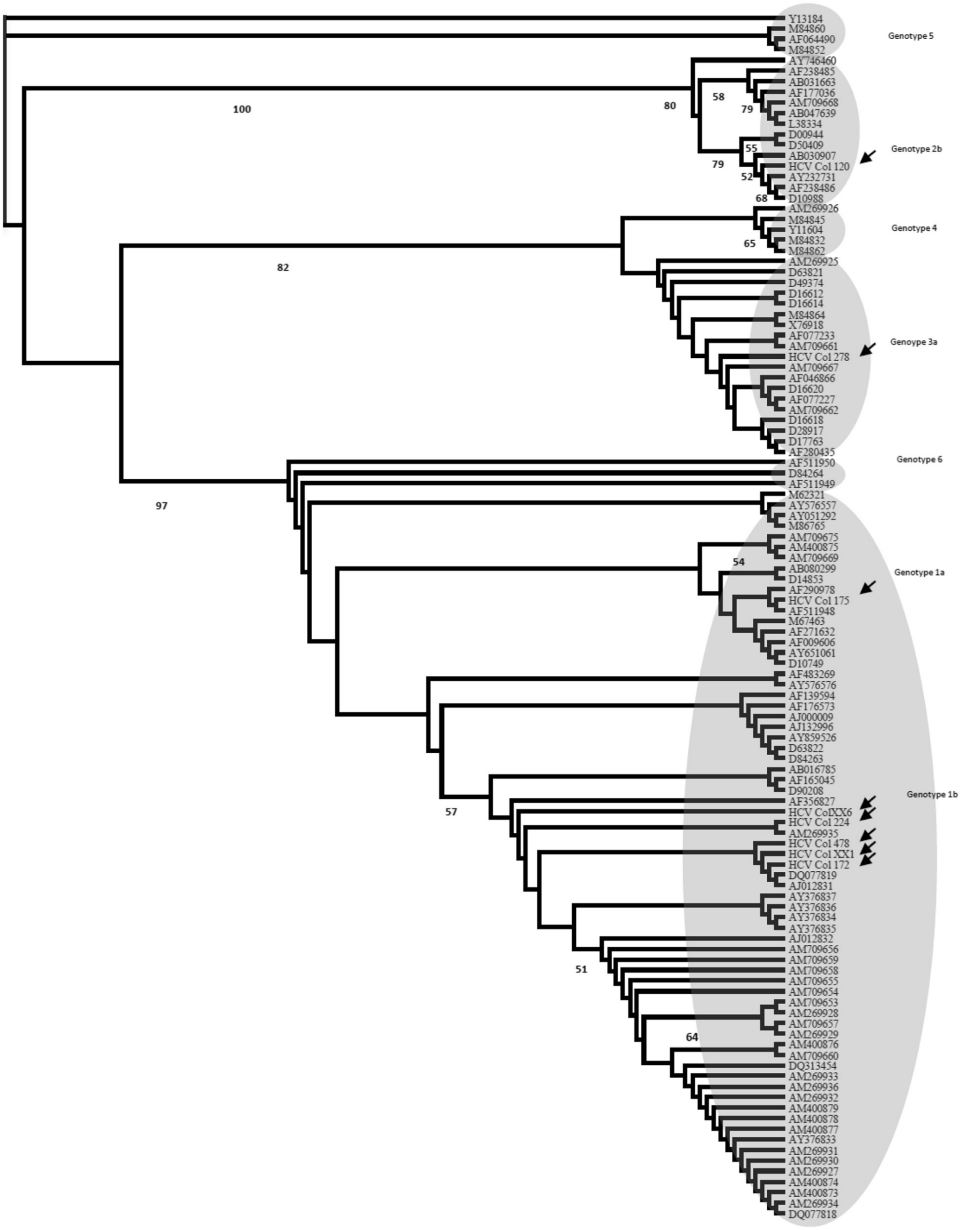
**Phylogenetic analysis of 5′UTR sequences of HCV strains.** Rooted phylogenetic tree generated with program PAUP and neighbor joining method. The arrows correspond to samples from multi-transfused patients of this study. Shaded areas indicate the expected clusters of sequences prototypes. The sequence of HCV genotype 5 was used as out-group (Y13184).

The phylogenetic tree using the neighbor-joining method showed the expected clades for the HCV prototypes (Figure 2). Isolates HCVCOL_175, 224, 120, 278 and XX6 were grouped into the same genotypes/subtypes previously designated by RFLP and BLAST. On the other hand, the isolates HCVCOL_172, 478 and XX1 were grouped in genotype 1 based on phylogenetic analysis. These results suggest a limitation of the 5’UTR RFLP-based genotyping method to discriminate the pattern of some strains of genotype 1. The inferring consensus tree showed a similar topology; indeed, the clade of genotype 1 was supported by high bootstrap values. This finding was confirmed by maximum parsimony analyses (data not shown).

**Figure 3:**
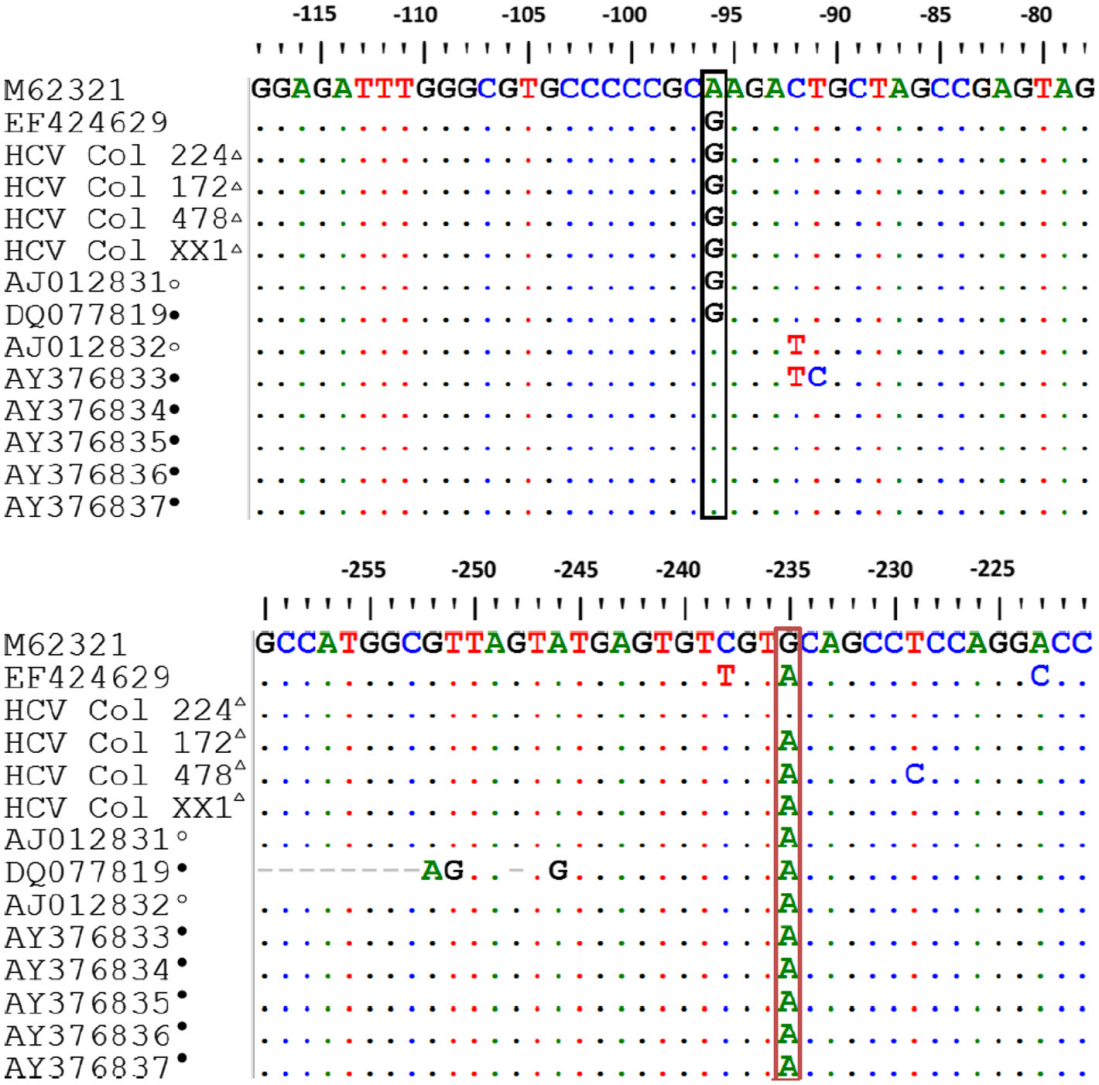
**Alignment of HCV 5′UTR sequences from Colombian multi-transfused patients.** M62321: sequence genotype 1a USA; EF424629: sequence genotype 6 Taiwan. Colombian multitransfused HCV ∆; Uruguay HCV ○ sequences; Argentina HCV ● sequences.

To assess the differences observed between the techniques used to identify the HCV genotypes, the sequences of the strains HCVCOL_172, 478, XX1 were aligned with HCV sequences previously reported in patients from Argentina and Uruguay (Figure 3). The Colombian isolates with the unexpected RFLP pattern were grouped into one sub-clade together with isolates of genotype 1, subtype 1b from Argentina and Uruguay [20]. Based on the HCV genome sequence described by Choo et al. [3], two unique substitutions were found; these mutations were localized at nucleotides −96 and −235. The former substitution corresponds to the change from adenine to guanine (A96G) and the second one to the transition G235A. When the substitutions were compared with the sequences recognized by each restriction enzyme used in the RFLP assay, a polymorphism (GTGC to GTAC) was detected that correlates with the substitution G235A generating a restriction site for the enzyme *RsaI* (GTGC to GTAC)*,* modifying the RFLP pattern of genotype 1. This polymorphism had been previously described in samples obtained from patients with chronic HCV infection in Argentina and Uruguay [21].

On the other hand, relevant substitutions were observed at positions −96 and −135 positions (G96A, C135A) in some strains, as well as an insertion of adenine at nucleotide −66 (66A) in one isolate (data not shown). All these mutations were located in domain III of the Internal Ribosome Entry Site (IRES), a secondary structure of the viral genome that mediates recognition by the 40S ribosomal subunit and the binding of the translation initiation factor 3 (eIF3). Further studies are necessary to determine the biological consequences of these mutations [22-24].

Based on the results of the phylogenetic analysis and/or RFLP, the circulation of HCV genotype 1, subtypes 1b (8/12, 66.7%) and 1a (2/12, 16.6%), genotype 2, subtype 2b (1/12, 8.3%) and genotype 3, subtype 3a (1/12, 8.3%) was confirmed in this high risk population (Table 1).

A number of studies were carried out previously to describe the HCV distribution in donors and patients with chronic liver diseases in Colombia [18,25-28]. Subtype 1b was the most frequently found in all these studies, although only two such studies correspond to phylogenetic analyses of HCV samples. In the first study, Mora et al. [18] explored the HCV genotype distribution among 53 samples from blood donors. As described in other South American countries, subtype 1b was the most prevalent in this donors population (82.8%); interestingly, this prevalence is one of the highest described among different studies performed worldwide. The distribution of the other subtypes and genotypes in this population donor was 1a 5.7%, 2a 5.7%, 2b 2.8%, and 3a 2.8%. The second study corresponds to the description of HCV genotypes in samples of liver tissue obtained from patients with end-stage liver diseases. The strains were grouped into subtypes 1a (one sample) and 1b (three samples) [29].

As mentioned above, HCV subtype 1b is mostly found among persons with a history of blood transfusion and among older individuals. Moreover, several studies have linked HCV subtype 1b to patients with bleeding disorders [30,31]. In the present study genotype 1 was the only genotype characterized in patients with hemophilia: five patients were infected with subtype 1b and two patients had subtype 1a (Table 1). The five patients in other diagnostic categories (hemodialysis, acute bleeding or oncologic illnesses) were infected with three different genotypes/subtypes, 1b (3 patients), 2b (1 patient) and 3a (1 patient) suggesting a more diverse origin of their infections.

Furthermore, seven isolates of subtype 1b characterized in our study were obtained from patients who had received their first transfusion before 1986. HCV screening of blood donors became mandatory in Colombia in 1993; but its universal coverage was not reached until 1997 [32].

In fact, Mora et al. [18] estimated that HCV subtype1b was introduced in Bogota around 1950 and spread exponentially in the period 1970 to 1990 probably carried out by blood transfusion. As described by Romano et al. [33] in Brazil, subtype 1b was the first HCV subtype introduced in Sao Paulo that possibly spread by blood transfusion and unsafe medical practices. Subtype 1b was followed by subtype 3a, and finally subtype 1a was the last subtype to emerge.

Subtypes 1a and 3a have been related to IDU in some studies [10-13]. However the predominance of subtype 1a found among hemodialysis patients in Brazil and the risk of infection related with the length of the time on hemodialysis suggested nosocomial HCV transmission [34]. In the present study subtype 1a was found in two samples from patients with hemophilia, whereas subtype 3a was detected in one hemodialysis patient.

This dynamics of HCV was recently also demonstrated in a cohort of repeat donors in the USA in the period January 2006 through December 2009. Subtype 1a was the most frequent HCV genotype in prevalent and incident cases; subtype 3a strains were significantly more frequent in incident cases whereas subtype 1b was less frequent in incident than in prevalent cases [8]. These results suggest that subtypes 1a and 3a have emerged recently and are spreading rapidly in some countries [10-13].

Additional studies and analysis of others HCV genome regions should be conducted in order to establish the phylogenetic relationship among HCV sequences obtained from Colombian blood donors, patients with chronic liver disease and multi-transfused patients [35]. The analysis of HCV sequences by genotype, clinical status, year of isolation and geographic origin could provide evidence of the HCV infection pattern spread in Colombia.

In conclusion, this is the first report on HCV genotypes among multi-transfused patients in Colombia where subtype 1b was the most prevalent. The mutation G235A in the 5′UTR of three HCV isolates generated an additional restriction site and, therefore, an RFLP pattern different from those previously described for genotype 1. Although the number of samples analyzed is relatively small, the results of this study have interesting implications regarding the HCV genotypes in a population exposed to blood transfusion during the time when TTI represented a serious health problem in Colombia. Molecular epidemiology studies are very important to understand the transmission dynamics of HCV infection in Colombia and in Latin America.

## Methods

### Study population

Five hundred multi-transfused patients were recruited at the Pan American Health Organization (PAHO) multi-center study between February and September 2003 in four hospitals of the two largest cities in Colombia, Bogota and Medellin. The present study was carried out with samples from 45 anti-HCV positive patients of this cohort.

The serum samples were stored at −70°C in air tight vials with plug seal caps, since their collection in 2003, and not thawed until immediately before the analysis.

### Ethics statement

All procedures adopted in this study were followed the terms established by the Ethics Committees of PAHO, Universidad de Antioquia, Instituto Nacional de Salud and Instituto Nacional de Cancerologia. The terms of informed consent were signed by all patients.

### Molecular detection of HCV

Total RNA was extracted from samples using TRIzol LS Reagent (Invitrogen). The 5 HCV ′UTR was detected by RT-nested PCR, as described by Chan et al. [36]. For cDNA synthesis, the viral RNA was reverse transcribed using primer 209 and Moloney Murine Leukemia Virus reverse transcriptase (Invitrogen). The 5′UTR was amplified by nested PCR using primers 209 and 939 and primers 940 and 211 [36]. The amplified products were analyzed by agarose gel electrophoresis.

### RFLP analysis and sequencing for genotype identification

To determine the HCV genotype, RFLP analyses were performed as described by Davidson et al. [37] with some modifications. Briefly, the amplified products were digested in separate reactions with the restriction enzymes *RsaI, HinfI* or *MvaI* at 37°C for 1 hour, and thereafter with *BstUI* at 60°C for 1 hour. Two cycles of enzyme denaturation were then performed at 95°C for 10 min. The digested products were analyzed by polyacrylamide gel electrophoresis. The genotype was determined by the pattern of each sample and compared with the predicted restriction patterns of 50 HCV sequences available in GenBank, using the BioEdit 7.9.0.0 software (lbis Biosciences, Canada).

### Phylogenetic analysis of the HCV 5′UTR

Phylogenetic analysis of the 5′UTR sequences was carried out to identify the clade and subtype distribution. The amplified 5′UTR fragment was purified and sequenced (Macrogen, Korea) using the automated dideoxynucleotide method (BigDyeTM terminator). A total of 106 prototype HCV sequences available in GenBank were included in the analysis. Rooted neighbor-joining and maximum parsimony trees were constructed using the PAUP 4.0 (Phylogenetic Analysis Using Parsimony) and the Mega 4.1 (Molecular Evolutionary Genetics Analysis) software. Reliability of the trees was evaluated statistically by bootstrap analyses with 100 and 1000 replicates, respectively.

### Pair-wise comparison of HCV isolates

HCV samples that exhibited no identifiable pattern by RFLP were sequenced and subjected to pair-wise comparisons. The sequence of each HCV sample was aligned with reference sequences using the BioEdit 7.9.0.0 software. Genbank accession numbers of reference sequences: Argentina isolates (DQ077819, AY376833, AY376834, AY376835, AY376836, AY376837), Uruguay isolates (AJ012831, AJ012832), and prototype sequences (M62321, EF424629, HCVCOL_224) of the genotypes 1a, 1b and 6, respectively.

## Abbreviations

HCV: Hepatitis C virus; RFLP: Restriction fragment length polymorphism; 5′UTR: 5′untranslated region; RT-PCR: Reverse transcriptase- polymerase chain reaction; IRES: Internal ribosome entry site; NJ: Neighbor-joining; MP: Maximum parsimony; BLAST: Basic Local Alignment Search Tool; cDNA: Copy DNA; ELISA: Enzyme-linked immunosorbent assay; TTI: Transfusion-transmissible infections; IDU: Intravenous drug users; eIF3: Eukaryotic initiation factor 3.

## Competing interests

The authors declare that they have no competing interests.

## Authors' contributions

Conceived and designed the experiments: MCN, FCM. Performed the experiments: DdF, FCM. Analyzed the data: MCN, FCM, DdF, MB, MPA, SJ, GC, JCR. Wrote the paper: MCN, DdF, FC. All the authors have read and approved the final manuscript.
